# Rhinitis in the geriatric population

**DOI:** 10.1186/1710-1492-6-10

**Published:** 2010-05-13

**Authors:** Jayant M Pinto, Seema Jeswani

**Affiliations:** 1 Section of Otolaryngology-Head and Neck Surgery, Department of Surgery, The University of Chicago, Chicago, IL, USA

## Abstract

The current geriatric population in the United States accounts for approximately 12% of the total population and is projected to reach nearly 20% (71.5 million people) by 2030[1]. With this expansion of the number of older adults, physicians will face the common complaint of rhinitis with increasing frequency. Nasal symptoms pose a significant burden on the health of older people and require attention to improve quality of life. Several mechanisms likely underlie the pathogenesis of rhinitis in these patients, including inflammatory conditions and the influence of aging on nasal physiology, with the potential for interaction between the two. Various treatments have been proposed to manage this condition; however, more work is needed to enhance our understanding of the pathophysiology of the various forms of geriatric rhinitis and to develop more effective therapies for this important patient population.

## Classifications

Rhinitis is defined as inflammation of the nasal mucosa and is characterized by symptoms of congestion, rhinorrhea, itching of the nose, postnasal drip, and sneezing[2]. In the geriatric population, a broad interpretation of this symptom complex may also include crusting within the nose, cough, excessive drainage, olfactory loss, and nasal dryness[3,4].

Rhinitis can be divided broadly into two major categories: allergic and nonallergic (Appendix 1).

Allergic rhinitis is an IgE-mediated inflammation of the nasal passageways triggered by various allergens such as dust, pollens, or molds. Symptoms of allergic rhinitis may be classified as seasonal or perennial. An international working group modified this classification scheme due to potential difficulties in differentiating between seasonal and perennial symptoms and created the Allergic Rhinitis and its Impact on Asthma (ARIA) Report[5]. The ARIA guidelines temporally classify allergic rhinitis as 'intermittent' if symptoms are present less than four days per week or less than four consecutive weeks, or as 'persistent' if symptoms are present more than four days per week and for more than four consecutive weeks. Severity of symptoms is graded as 'mild' if they are present but not troublesome, and as 'moderate/severe' if they lead to sleep disturbance, impairment of daily activities, or impairment of school or work.

Nonallergic rhinitis is characterized by non-IgE-mediated symptoms typical of rhinitis, such as congestion and clear rhinorrhea, with less prominence of sneezing and ocular/nasal pruritis[6,7]. The associated symptoms may be perennial or sporadic, lacking a clear seasonality, and may be exacerbated by nonspecific triggers such as odors, food, emotion, or change in atmospheric conditions[5,8,9]. Though no formal classification system exists, nonallergic rhinitis can be further subcategorized; most commonly seen in older patients are the vasomotor, atrophic, gustatory, and medication-induced subtypes[10,11].

## Epidemiology

Allergic rhinitis affects approximately 10-30% of American adults[2,12]. The condition predominantly affects males in their late teens or young adulthood and the prevalence decreases with age[12,13]; yet, it is estimated that three per one thousand individuals over the age of 65 also suffer from allergic rhinitis with a shift to female predominance after adolescence[13,14]. Cross-sectional and longitudinal studies have shown that both allergic rhinitis symptoms and allergic skin test sensitivity become milder over time; however, these findings may not necessarily correlate[15,16]. Such changes may be due to alterations in immune function with age[17,18]. For instance, total IgE levels and eosinophil degranulation in response to cytokine stimulation decrease with age[19,20]. Furthermore, repetitive exposure to allergens may induce tolerance or anergy over time through mechanisms that are not completely clear[14].

There is substantially less research regarding frequency of nonallergic rhinitis in comparison to allergic rhinitis in older subjects. An estimated 19 million people in the United States suffer from nonallergic rhinitis[21]. The prevalence of nonallergic rhinitis is greater in females and the incidence of diagnosis increases with age[22-24]. Greater than 60% of rhinitis patients over the age of 50 suffer from a nonallergic etiology[9].

## Effects of Rhinitis on Quality of life

Several studies have shown the deleterious effects of rhinitis on the quality of life in symptomatic patients. Benninger et al found that allergic rhinitis can result in significant sleep disturbance and fatigue using the Rhinosinusitis Disability Index (RSDI), a validated outcomes tool that assesses how allergic rhinitis affects quality of life[25]. Complaints of poor sleep are already common among older individuals due to various sleep disorders as well as the normal aging process[26], thus allergic rhinitis may exacerbate these problems. Lack of sleep can alter physiological processes such as glucose metabolism, cognition, appetite control, and endocrine function, all critical physiologic processes in older people[27,28]. Longitudinal studies have demonstrated that sleep complaints in the geriatric population are also associated with lower self-reported health status, depression, and increased mortality[29-32]. Other effects of allergic rhinitis include headache, poor concentration, and general irritability. These symptoms may hinder an individual's ability to carry out physical and social responsibilities effectively[2]. Both of these domains were found to be large factors that contribute to geriatric quality of life[33,34].

Little data are available that specifically address the effects of nonallergic rhinitis on quality of life, especially in geriatric subjects. Because allergic and nonallergic rhinitis share similar symptoms, the two conditions are perceived by patients similarly[35] and it would be plausible to extrapolate data on allergic rhinitis to model the effects of nonallergic rhinitis. In fact, a recent study demonstrated a decrease in health-related quality of life in both allergic and nonallergic rhinitis patients; indeed, there was no significant difference in the degree of impairment between the two patient populations[36].

## Physiological Changes with age that may affect Rhinitis

### Immunosenescence

Rhinitis is an inflammatory disease, as such, mechanisms and presentation of the condition are altered as immune function changes with age, a concept entitled immunosenescence. A critical component of the immune system is the thymus, which rapidly involutes from adolescence to near middle age, followed by an approximate 1% loss per year thereafter[37]. The decline in functional mass causes depressed production of naïve T-cells leading to impaired cell-mediated immunity[37,38]. Despite thymic involution, the total T-cell pool remains constant due to an increase in production of memory T-cells[38]. The cause of this heterogeneity in the lymphocyte pool remains unknown. With the aging process also comes decreased T-cell responsiveness to growth factors, altered lymphocyte response to specific antigens, and diminished IL-2 production and receptor expression[17]. An imbalance in the Th1/Th2 ratio occurs during immunosenescence, with a shift towards Th2, leading to further altered cytokine production[39]. This is somewhat of a paradox since the incidence of allergic rhinitis declines with age. The diminished T-cell response may be associated with the increased incidence of malignancy and infections in the geriatric population[17,40], whereas the aberration in cytokine production and inflammatory response may explain chronic or late onset rhinitis.

B-cell function changes with age as well. Although the peripheral B-cell population remains consistent, there is less IgG isotype class switching, and the number of antigen-specific antibodies decreases while the number of autoantibodies and circulating immune complexes increase[17,18]. This may explain the fact that older individuals are prone to infection, have decreased immune response to vaccines, and have increased prevalence of autoimmune diseases[17,38,40]. These changes might also contribute to the milder symptoms as well as the decreased incidence of allergic rhinitis in the geriatric population.

### Changes of the Aging Nose

#### Structural

As individuals age, several changes in nasal anatomy and physiology occur which may affect the development and expression of rhinitis. A loss of nasal tip support develops because of weakening of fibrous connective tissue at the upper and lower lateral cartilages[4]. Collagen and elastin loss, maxillary alveolar hypoplasia, and decreased facial musculature lead to a drooped tip[41]. Furthermore, weakening and fragmentation of septal cartilage and retraction of the nasal columella leads to changes in the nasal cavity[42]. A combination of these structural changes may decrease nasal airflow leading to complaints of nasal obstruction commonly seen in geriatric rhinitis patients.

#### Mucus

Mucosal epithelium atrophies with age and older patients are frequently dehydrated[43,44]. These factors may account for the excessively thick mucus in older patients. Thickened mucus along with decreased mucociliary clearance (see below) is thought to lead to common rhinitic complaints such as postnasal drip, cough, and globus. Edelstein was able to demonstrate that the prevalence of postnasal drip, nasal drainage, coughing, and sneezing increased with age[4].

#### Nasal Humidification and Dryness

It is well recognized clinically that older people are more susceptible to dryness in the nose. Lindemann et al illustrated that temperature and humidity values in the nasal cavities were significantly lower in geriatric patients when compared to younger individuals[45]. Other reasons for decreased humidification include age-related changes in nasal vasculature. For instance, submucosal vessels become less patent and therefore are not able to moisten and warm inspired air in the older nose as compared to the younger nose[44]. These findings in geriatric patients likely explain the typical symptoms of nasal irritation related to dryness and crusting.

#### Nasal Airflow

The effects of age on nasal airflow still remain largely unclear. Calhoun et al did not find a relationship between age and nasal resistance[46], whereas Vig and Zajac determined that there is a direct relationship between age and both nasal resistance and breathing mode[47]. Edelstein found a significant correlation between aging and nasal airway resistance, before and after decongestant use[4]. Kalmovich et al studied endonasal architecture in geriatric patients using acoustic rhinometry and concluded that endonasal volumes and minimal cross-sectional areas gradually increase with age[48]. The reason for the discrepancy between the latter two studies is unclear. Sahin-Yilmaz and Corey suggest this difference may be due to decreased functioning of the nasal mucosa[10]. The authors note that the estrogen content in the nasal mucosa decreases with age and can subsequently cause to loss of softness and elasticity, leading to increased airway resistance. Post-menopausal women may also suffer from olfactory loss, nasal congestion, and an increase in mucociliary time secondary to hormonal changes[49]. Estrogens modulate mucosal function by modifying the local concentration of neurotransmitters or their receptors, which regulate basal vasculature and glandular secretions[49]. Recent evidence suggests that the number of specific estrogen receptors (ERβ) within the nasal mucosa positively correlates to rhinitic symptoms, yet the mechanism of the receptors' effects on nasal mucosa is yet to be elucidated[50]. It is plausible that other airflow abnormalities could also underlie nasal complaints in older subjects.

#### Mucociliary Clearance

Studies show that the frequency at which cilia beat, as well as time for mucociliary clearance within nasal epithelium, slow with age[51]; however, the number of ciliated cells in the nasal epithelium does not change[4]. In fact, Kirtsreesakul et al have recently shown that the severity of symptoms was significantly correlated with the mucociliary transport time in patients classified with moderate-severe allergic rhinitis[52]. This may be due to poor clearance of allergen and irritants, as well as stasis of thick, dehydrated mucus within the nasal cavities and nasopharynx, leading to rhinitis complaints among the geriatric population of postnasal drip, cough, and globus.

#### Olfaction

Olfactory function decreases with age, with more rapid decline after the seventh decade. Both the sense of smell (detection) and the ability to discriminate between two scents is decreased with the normal aging process[53]. Olfactory dysfunction is also commonly associated with rhinitis. One study demonstrated that 71% of study subjects complaining of dysosmia had positive allergy skin tests[54]. The mechanism for olfactory dysfunction in allergic rhinitis patients has been typically attributed to nasal obstruction; however, newer data suggest that the etiology may be due to inflammation in the olfactory cleft itself[55]. This inflammation may be responsive to intranasal steroids. Of note, one trial has demonstrated that the sense of smell in nonallergic rhinitis patients was worse than in allergic rhinitis patients[56]. Together, this data illustrates that though olfactory dysfunction may be primarily due to the aging process, rhinitis may present as a 'second hit' that aggravates the problem.

## Pathophysiology and Clinical Presentation of Rhinitis

### Allergic

To briefly review, allergic rhinitis is the result of type I hypersensitivity reactions whereby exposure to allergens in susceptible individuals leads to sensitization by production of specific IgE antibodies directed against these extrinsic proteins. This antibody then binds to the surface of mast cells, and when the allergen is reintroduced, IgE cross-binding to the antigen leads to mast cell degranulation[57]. Within seconds of contact, inflammatory mediators such as histamine, leukotrienes, and prostaglandin D2 are released causing vascular endothelial dilation, which subsequently causes leakage and mucosal edema[58,59]. This leads to nasal obstruction and symptoms of congestion, redness, tearing, swelling, ear pressure, and postnasal drip. Irritant receptors are stimulated by the allergen causing itching and sneezing[60].

Within four to eight hours of initial exposure, cytokines attracted by previously released mediators lead to recruitment of other inflammatory cells to the mucosa, such as neutrophils, eosinophils, lymphocytes, and macrophages[59]. The inflammation persists and this stage is termed the late-phase response. The late-phase response presents similar to the early phase, however, sneezing and itching are less prominent, whereas congestion and mucus production are more severe. The late phase may persist for hours or days[61].

Though its peak incidence is during young adulthood, allergic rhinitis is prevalent among older people. In fact, the 2005 National Center for Health Statistics report stated that 10.7% of individuals between 45-64 years of age, 7.8% of patients 65-75 years of age, and 5.4% of patients older than 75 are affected by allergic rhinitis[62]. Along with the anatomic and physiologic changes of the nose, non-specific immune changes such as decreased mucus production and ineffective cough mechanisms are all thought to contribute to persistent or late-onset allergic disease in older people, as these processes are necessary for clearance of allergens and irritants[17]. Interestingly, Jackola et al. illustrated that atopic individuals with a positive family history did not have a change in severity or sensitivity of atopy as they age. Also, there were no changes in the amount of IgE specific to ragweed extract, despite age-related decline of total serum IgE[63]. Mediaty et al also demonstrated that immunosenescence does not affect increased IgE levels in atopic patients with atopic dermatitis or high serum IgE levels[64]. In summary, these findings suggest that the atopic predisposition remains present into advanced age in these subgroups of patients. Therefore, allergic rhinitis should not be overlooked in the geriatric population if a patient's history and symptoms are consistent with this condition.

### Nonallergic

#### Vasomotor

Vasomotor rhinitis is the most common form of nonallergic rhinitis, and its prevalence is increased in the aging population[22]; however, due to the difficulties in classifying this condition, epidemiological data is scant. This condition does not have an obvious immunologic or infectious etiology and is not associated with nasal eosinophilia[2]. Prominent symptoms of vasomotor rhinitis include nasal obstruction, rhinorrhea, and congestion[6,65]. These symptoms are exacerbated by strong odors or fumes, bright lights, and changes in weather or humidity[65,66].

The mechanism of vasomotor rhinitis is unclear. One theory is that autonomic instability may lead to both hyperactive parasympathetic activity and an imbalance in sympathetic to parasympathetic activity in vasomotor patients, which cause nasal congestion and rhinorrhea[67]. The sympathetic pathway promotes nasal airway patency by secretion of norepinephrine and neuropeptide Y[68], whereas the parasympathetic pathway releases substances that lead to congestion and mucus secretion such as acetylcholine. Thus, vasomotor rhinitis in older patients may represent a decline in control of neurological responses that affect nasal physiology.

Another possibility may be that neurogenic reflex mechanisms triggered by environmental factors (e.g., ozone, cigarette smoke) lead to inflammatory responses in the nose. Sensory nerves of the nasal mucosa respond to chemical stimuli by initiating sneezing and nasal hypersecretion through reflex pathways. The sensory fibers that are unmyelinated and slow conducting belong to the C-fiber type and contain neuropeptides such as substance P, calcitonin gene-related peptide, and vasoactive intestinal peptide, which regulate glandular secretions and vascular tone[67,69]. Baraniuk and colleagues have demonstrated that these neuropeptides communicate with immune mediators, such as histamine. The interplay between these molecules may cause neural responses and vice versa, all leading to an integrated response to external stimuli[70]. These mechanisms may be involved in the etiology of vasomotor rhinitis in older people.

Cardell et al recently studied nasal mucosa biopsies of nonallergic rhinitis patients using microarray analysis[71]. The group noted at least ten genes to be involved in nonallergic rhinitis, relating to functions of cellular movement, hematological system development, and immune response. Two of these genes, *c-fos *and *cell division cycle 42 *(*cdc42*) were found to have pivotal roles in the possible mechanistic pathways of nonallergic rhinitis and the authors believe these genes could be potentially useful as biomarkers for this condition and aid in diagnosis. These data are preliminary and will require follow-up.

#### Gustatory

Gustatory rhinorrhea is characterized by profuse watery rhinorrhea following ingestion of certain foods. These symptoms can be socially awkward and even lead to decreased nutrition. Commonly, alcohol and spicy or cold foods are the culprits. With spicy food, the capsaicin content induces neuropeptide release from sensory nerve fibers, leading to overstimulation of the parasympathetic nervous system[67]. Baraniuk and colleagues demonstrated the significance of the TRP (transient receptor potential) receptor family in the regulation of sensory neuron depolarization and repolarization, glandular exocytosis, and many other functions. Substrates for these receptors include capsaicin, extremely high or low temperatures, alcohol, mustard oil, and some garlic components[72]. One speculation is that TRP receptors may play a role in gustatory rhinorrhea.

#### Medication-induced

Several classes of medications are known to induce rhinitis (Appendix 2). The mechanisms causing this include alteration of autonomic regulation on nasal mucosa and vasculature, platelet activity, immune effects, and hormonal effects. This condition is of particular importance in older patients, as polypharmacy has become a common issue among the geriatric population with an increasing amount of comorbid conditions. Although the number of individuals over 65 years of age represents less than 15% of the total population, this group accounts for over one third of prescription drug use nationwide[73]. Furthermore, Kaufman et al discovered that 57% of American women greater than 65 years of age use at least five medications and 12% use at least ten medications[74]. Common agents used in the geriatric population that can induce rhinitis are discussed below.

Medications with effects on the cardiovascular system carry side effects of rhinitis due to disruption of the normal sympathetic tone that causes vasoconstriction of local vessels. Medications such as alpha and beta-blockers, centrally acting anti-hypertensives, and angiotensin converting enzyme (ACE) inhibitors that inhibit sympathetic tone lead to vasodilation and symptoms of nasal congestion. Antipsychotics also have well-known rhinologic side effects due to their alpha and beta blocking properties[75].

Topical decongestants can cause rebound vasodilation with overuse. The older population is at increased risk for this adverse effect due to thinning and dryness of their nasal mucosa[76].

Aspirin-sensitive patients may suffer from rhinitis with use as well as prolonged epistaxis due to its anti-platelet activity. Other systemic medications that cause rhinitis are contraceptives, erectile dysfunction therapies, immunosuppressants, antivirals, penicillamine, and oral retinoids[75].

#### Primary atrophic

Geriatric rhinitis, or primary atrophic rhinitis, is an imprecise term used to signify rhinitis due to the age-related changes in nasal physiology (nasal glandular atrophy, vascular changes, decreased nasal humidification, decreased mucociliary clearance, and structural changes of the nose)[77]. Histopathological changes associated with primary atrophic rhinitis include mucosal atrophy, squamous metaplasia, and chronic inflammatory cell infiltrate[78]. Garcia et al studied how these changes lead to symptoms using techniques of computational fluid dynamics of airflow and water and heat transport, finding that excessive evaporation of the mucus layer secondary to widened nasal cavities and decreased mucosal surface area are integral components of atrophic rhinitis[79]. These changes lead to thickened and persistent mucus and altered nasal airflow. Recent studies have attempted to elucidate the role of apoptosis in rhinitis, finding that nasal epithelium from patients suffering from atrophic rhinitis display increased activity of caspase 3, a key protein in the apoptosis cascade[80]. This finding directs future studies to investigate therapeutic strategies that may regulate apoptosis.

Patients suffering from primary atrophic rhinitis, a diagnosis of exclusion, typically present with symptoms of postnasal drip, chronic cough, and nasal obstruction and dryness. Patients may also complain of a frequent need to clear the throat, thick and dense secretions, as well as bothersome nasal crusting upon awakening[77,78,81]. Because this progressive condition presents with symptoms similar to other types of rhinitis, it is often improperly diagnosed and undertreated. More work needs to be done to recognize atrophic rhinitis as well as to administer effective treatment.

For completeness, we mention that secondary atrophic rhinitis is seen in patients with extensive nasal surgery, trauma, granulomatous diseases, and radiation therapy[82] and will not be discussed here.

## Evaluation

Diagnosis and treatment of rhinitis in the older population is complicated by comorbid conditions. Approximately 50% of people over the age of 75 have three or more diseases and take three or more medications[83]. Within this population, there are also concerns of compliance due to physical or cognitive impairments and financial issues[83]. Furthermore, many older patients with rhinitis complain of "sinus trouble" or "allergies", thus it is difficult to assess the specific type of rhinitis involved or the appropriate treatment[77].

Evaluation of an older patient with rhinitis should begin with a complete history. Details regarding length and timing of symptoms, exacerbating factors, and response to medications should be elicited from the patient. Also important for investigation are environmental exposures such as tobacco smoke, pets, pollution, housing type which may be older and might contain formaldehyde for insulation or upholstery finishing, cockroaches and rodents. Activities requiring use of latex gloves, certain cleaning products, certain glues, wood dust, and acid anhydrides can trigger symptoms of rhinitis[84]. Interestingly, psyllium powder from Metamucil preparation (commonly used in the older patient for constipation) has been reported to induce acute rhinitis[85].

Past medical history consisting of trauma to the nose or face, asymmetry of nasal breathing due to structural causes, allergic conditions such as asthma and eczema, as well as family history of atopy should be noted. Though the typical age of onset of allergic rhinitis is under age 20 years, humans can be sensitized at any time [14], therefore an allergic cause should not be perfunctorily dismissed.

Physical examination should begin with externally inspecting the tip of the nose for drooping and absence of structural support. Internally, the physician should assess nasal patency, turbinates, straightness of the septum, presence of polyps, and signs of inflammation. Most of this endonasal examination can be accomplished with an otoscope. Mucosal evaluation may be challenging to interpret as both allergic and nonallergic rhinitis can present with mucosal pallor, edema, or hyperemia[2]. Overuse of topical medications may cause the nasal mucosa to appear reddened. The quality of secretions may aid in distinguishing the etiology of rhinitis. For instance, allergic rhinitis typically presents as watery mucus, whereas in mucociliary defects or severe obstruction, thick mucus can be seen pooling on the nasal floor[2]. Furthermore, mucopurulent drainage along with "cobblestoning" of the pharynx may be suggestive of chronic rhinitis complicated by acute sinusitis. In most cases of rhinitis, findings should be bilateral. Unilateral findings may reflect anatomic pathology or neoplasm requiring further workup including nasal endoscopy or sinonasal imaging by computed tomography (CT) scan. The remainder of the physical examination should be employed to eliminate other causes of rhinitis including cerebrospinal fluid rhinorrhea and tumors.

Allergy testing is useful both to determine atopy status with total serum IgE (usually > 100 U/mL)[86] as well as to identify the specific offending allergens (specific IgE). It should be noted, however, that response to skin testing decreases with age and photo damage, requiring skin tests in geriatric patients to be interpreted with care[87]. Other factors that may affect a skin test in the geriatric population are medications (most notably long-acting antihistamines and tricyclic antidepressants), blood pressure, temperature of the extremity, and change in the allergen exposure over time[87]. When administering a skin test to an older individual, a sun-protected area of the skin should be used for testing, such as the lower back. If an acceptable area cannot be found, the physician should consider *in vitro *testing.

By definition, patients with nonallergic rhinitis demonstrate negative test results. Nonallergic rhinitis is a diagnosis of exclusion due to the absence of specific laboratory tests that confirm this diagnosis[67].

Adjunct testing can also be useful in the evaluation of rhinitis in older subjects. Upper airway endoscopy is valuable in identifying any anatomic abnormalities that may not have been visualized on anterior rhinoscopy, such as septal deviation, nasal polyposis, or mucosal atrophy. Moreover, the middle meatal sinus ostia can be assessed for signs of obstruction which could predispose to sinusitis[84,88]. Sinus imaging by screening CT scan provides information on any obstruction of the osteomeatal complex, and can also aid in identifying polyps, turbinate edema, or bony abnormalities such as concha bullosae[84]. However, given cost and radiation exposure, imaging is reserved for cases of lack of response to therapy, high index of clinical suspicion, or pre-operative assessment for use during endoscopic sinus surgery to delineate anatomy[89,90]. Additional specialized testing (e.g. acid reflux testing by pH probe, assessment of nasal volume by acoustic rhinometry) can be useful for evaluation of exacerbating factors, such as gastroesophageal reflux disease or decreased nasal patency in select settings[23,91,92].

## Management

### General measures

There are a variety of methods to treat rhinitis in older subjects. In the case of both allergic and nonallergic rhinitis, the simplest therapy is eliminating exposure to known allergens and/or irritants. It should be noted that some avoidance measures (mite dust covers, air purifiers, carpet removal) have not been shown to be effective in reducing symptoms and pose barriers for some patients regarding cost. Humidification with saline nasal irrigation has been demonstrated to be a safe and effective technique to reduce nasal dryness and help with the clearing of thick, tenacious mucus[93,94]. Mucolytic agents may also help clear thick mucus and provide symptom relief. Emollients have been shown to help with nasal crusting[10]. For example, Johnsen et al demonstrated that in patients suffering from nasal dryness due to low humidity, use of sesame oil significantly improved mucosal dryness, nasal stuffiness, and nasal crusting when compared to an isotonic sodium chloride solution[95]. The measures are generally safe and can be used adjunctively with other treatments.

### Allergic Rhinitis

Treatment of allergic rhinitis has three main components: avoidance of exposure to known allergens, pharmacotherapy, and immunotherapy.

#### Allergen avoidance

Some forms of allergen avoidance can be effective in management of allergic rhinitis; though proof in randomized trials has been difficult to generate, this is still a standard recommendation for patients. Staying indoors with the windows closed while pollens and molds are in their seasonal and daytime peaks can decrease disease burden. Other measures include regular vacuuming of carpet, removal of pets from home, and frequent washing of bedding. These factors are particularly relevant for the older patient since s/he may spend significantly more time indoors and therefore may be exposed to allergens such as dust mites and indoor molds more than outdoor allergens such as pollens. As such, they may face a perennial allergen challenge which is sometimes more difficult to control. Cost and practicality must govern recommendations in this area for costly and difficult measures (e.g., carpet removal, HEPA filters, etc.) given the lack of evidence for efficacy for these measures.

#### Pharmacotherapy

Second generation antihistamines are standard in the treatment of mild allergic diseases. These agents are effective in reducing symptoms of nasal and ocular pruritis, rhinorrhea, and sneezing, but do little in managing nasal congestion[2,96]. Second generation agents are safe in older rhinitis patients since they do not carry the risk of anticholinergic or alpha-adrenergic activity[57,76]. First-generation antihistamines should not be prescribed as they have numerous potential adverse effects on the central nervous system and interactions with other medications, which are more pronounced in the geriatric population[10,57,76]. For example, these medications can affect driving performance more than alcohol, perturb the normal sleep cycle, and markedly affect attention and cognitive performance[97,98], all of these factors being germane to the older patient.

Topical antihistamines, such azelastine, are good alternatives to the oral therapy and are approved for seasonal allergic rhinitis in the United States. Studies have proven equal efficacy to ebastine, cetirizine, loratadine, and terfenadine in terms of symptom reduction and may also improve nasal congestion more so than oral antihistamines[99]. Azelastine has been shown to be well tolerated in geriatric patients[100]. Typical adverse events include bitter taste, sedation, headache, and application site irritation[99,101]. Topical antihistamines have demonstrated greater efficacy when combined with intranasal steroids than either agent alone[102]. A new formulation of azelastine was developed to reduce the bitter taste associated with the medication. This new product is as effective as the older version with similar frequency and constellation of side effects[103,104].

Intranasal steroids have become first-line treatment for moderate to severe allergic rhinitis and effectively treat all symptoms of rhinitis[105]. A recent randomized controlled trial studied the effects of mometasone furoate nasal spray in patients older than 65 years of age suffering from perennial allergic rhinitis, showing it to be an effective treatment in this cohort[106]. Intranasal steroids are generally well tolerated by older patients[10,107]; however, they can aggravate nasal dryness, epistaxis, and mucosal crusting in geriatric patients[108]. Therefore, careful instruction in use with patients is critical along with close follow-up to examine the presence of these problems in the nose.

Topical and systemic decongestants are alpha-adrenergic agonists that significantly reduce nasal congestion, however they do not relieve symptoms sneezing, pruritis, and secretions[109]. Decongestants can be used with antihistamines if a patient presents with multiple rhinitis symptoms including congestion. Oral agents are avoided in those older patients with multiple comorbid conditions such as coronary artery disease, diabetes, hypertension, hyperthyroidism, narrow angle glaucoma, and symptoms of bladder neck obstruction[96,110,111]. Side effects from oral decongestants include palpitations, insomnia, nervousness, and irritability. Some patients may have trouble with urination and a decreased appetite[2]. The major side effect of topical decongestant overuse is rebound vasodilation and nasal dryness, as well as the potential for rhinitis medicamentosa with prolonged use[105,112].

Leukotriene receptor antagonists (e.g. montelukast, zileuton) decrease the inflammatory response in allergic rhinitis and limit symptoms of congestion, sneezing, and rhinorrhea[113]. These agents are weak as a monotherapy and are commonly used as an adjunct to antihistamine or intranasal steroid treatment[96,114]. Long-term data has not been reported to determine safety of leukotriene inhibitors in the older patients, yet these medications seem to be well tolerated in this population[10,115]. These agents primarily help with congestion and are particularly useful in asthmatics where they may have the double benefit of improving lower airway disease.

Intranasal cromolyn sodium can be effective in minimizing allergic rhinitis symptoms in refractory patients. This agent inhibits the degranulation of sensitized mast cells thereby preventing the release of mediators of the allergic response and inflammation[116]. Patients who are given nasal cromolyn sodium must be instructed to use it before an anticipated allergen exposure and to use it on a regular basis during the period of exposure[2]. Cromolyn may require two to three weeks of use before any benefit is experienced and should be used three to four times per day[105]. The medication is generally well tolerated and side effects are minimal[116]. Cromolyn can be good option in older patients that cannot tolerate antihistamines and decongestants, or with use of multiple medications due to its lack of drug interactions [102,116].

#### Immunotherapy

Immunotherapy is typically regarded as a last line therapy when patients continue to have moderate to severe allergic rhinitis symptoms despite pharmacologic intervention. Few studies have been conducted on the efficacy of immunotherapy in the geriatric population; however, the data thus far seems positive. Eidelman et al reported a favorable response to specific immunotherapy in patients greater than 60 years of age compared to controls aged less than 60[117]. Asero conducted a study assessing patients older than 54 years of age with monosensitization to birch pollen and ragweed[118]. The trial showed immunotherapy to be an effective treatment in healthy older individuals with short disease duration (<10 years) whose symptoms cannot be adequately controlled by drug therapy alone. Further studies need to be performed to address the safety profile of this treatment within the geriatric population.

### Nonallergic rhinitis

#### Vasomotor rhinitis

##### Pharmacotherapy

Azelastine has been approved by the Food and Drug Administration (FDA) for treatment of vasomotor rhinitis. This agent has demonstrated anti-inflammatory effects and thus can be given for nearly all symptoms of vasomotor rhinitis including rhinorrhea, sneezing, postnasal drip, and congestion[119,120]. Intranasal steroids may be given for complaints of nasal obstruction or congestion[121]; however, a recent study has shown that steroid use for symptoms of vasomotor rhinitis triggered by temperature or weather may not be effective[122]. The use of anticholinergics in this condition is mainly for symptoms of rhinorrhea[6,123] but the applicability of this agent to geriatric patients has not been examined. Sneezing and congestion associated with vasomotor rhinitis may be relieved with cromolyn sodium, but is not as effective as a first-line treatment for these symptoms[6]. There is no evidence for use of topical or oral decongestants. Empiric use of decongestants is possible provided that the geriatric patient has no contraindications. In summary, pharmacotherapy for vasomotor rhinitis is symptom based, thus if one medication fails current practice is therapeutic trials with other listed agents.

##### Novel therapies for VMR

A Chinese group demonstrated polysaccharide nucleic acid fraction of the Bacillus Calmette-Guérin vaccine (BCG-PSN) to be a safe and effective treatment of vasomotor rhinitis with no reported adverse events[124]. This therapy requires multiple injections, which may be difficult and painful of older patients, and may also require adjunctive treatment with a topical antihistamine. Follow up for this trial was only six months and therefore more evidence is necessary for long-term efficacy and safety of BCG-PSN. Further study of these findings is needed before this can reach clinical practice.

#### Gustatory Rhinorrhea

Atropine decreases the parasympathetic response and thus is useful in treatment of gustatory rhinorrhea[67]. Intranasal anticholinergic agents, such as intranasal ipratropium, are FDA approved for use in rhinorrhea in allergic and nonallergic perennial rhinitis and are thus very effective for gustatory rhinorrhea if used before eating[125]. These medications have few local side effects such as epistaxis and nasal dryness[105].

Botulinum toxin is a newer therapy for gustatory rhinorrhea, however optimal site of administration, optimal dose, long-term efficacy, and side effects have yet to be determined[126,127].

#### Atrophic Rhinitis

The focus of treatment in atrophic rhinitis is to increase moisture content in the nose. This can be done with gentle hydration, nasal irrigation, improving mucus function with agents such as guaifenesin, or use of home humidifiers[108]. Over-the-counter mucolytic agents such as Alkalol can also provide some therapeutic benefit[128].

### Surgery

Surgical treatment is also an option in the geriatric population. First, structural causes can be addressed. Nasal reconstruction can be performed to reverse the aging effects on the nose, such as raising the nasal tip and supporting the lateral cartilage[10]. This should aid in air flow and nasal function. Similarly, septoplasty with or without inferior turbinate reduction has been shown to be beneficial in patients 65 years of age and older[129]. Moreover, functional endoscopic sinus surgery can be used to address concomitant sinus disease. Sinus surgery has been shown to be a safe and efficacious procedure in older patients[130,131] with improvement in quality of life. Reh et al demonstrated that older individuals with chronic rhinosinusitis had a similar degree of improvement in endoscopic and quality of life measures after endoscopic sinus surgery when compared to matched younger controls[132]. Surgery could be an effective treatment for an older individual, but the functional status of the geriatric patient must be assessed pre-operatively to determine if surgery is a suitable option.

## Conclusions

Rhinitis is clearly an important burden in the older community that needs to be further addressed as the geriatric population rapidly expands in the United States. The structure and function of the aging nose may contribute to the manifestations and mechanisms of this condition. This broad set of symptoms fall under a heterogeneous group of disorders, and thus the focus of therapy must first be classification of the patient within the proper subtype, and then engagement of appropriate therapy that is both safe and efficacious in older individuals. Treatment is challenging as little data exists on clearly beneficial treatments for several of these subtypes. Trial and error may prove to be useful in instances where no validated data are available for geriatric patients. Furthermore, use of adjunct measures such as allergen/irritant avoidance and nasal humidification are not only cost-effective, but can also limit the extent of polypharmacy in a population with multiple comorbid conditions. More research is needed to develop effective treatment protocols for this group of rhinitis patients.

## List of Abbreviations

1) ARIA: Allergic Rhinitis and its Impact on Asthma; 2) RSDI: rhinosinusitis disability index; 3) cdc42: cell division cycle 42; 4) TRP: transient receptor potential; 5) ACE: angiotensin converting enzyme; 6) CT: computed tomography; 7) HEPA: high-efficiency particulate air; 8) BCG-PSN: polysaccharide nucleic acid fraction of the Bacillus Calmette-Guérin vaccine; 9) FDA: Food and Drug Administration

## Competing interests

JMP has received investigator-initiated research funding from Schering Plough Corporation. SJ declares no competing interests.

## Authors' contributions

JMP conceived of the structure and content of the manuscript and assembled relevant references. SJ prepared the draft of the manuscript under the direction of JMP. JMP edited and revised the manuscript. Both authors read and approved the final manuscript.

## Author information

JMP is Assistant Professor of Surgery in the Section of Otolaryngology-Head and Neck Surgery at The University of Chicago. He directs the Program in Transitional Rhinology Research http://surgicalresearch.bsd.uchicago.edu/faculty/pinto/.

## Appendix 1 - Types of Rhinitis

Allergic

Seasonal

Perennial

Intermittent

Persistent

Nonallergic

Idiopathic/Vasomotor

Nonallergic rhinitis with eosinophilia syndrome (NARES)

Atrophic

Infectious

Drug-induced

Gustatory

Hormonal

Occupational

Granulomatous

Autoimmune

## Appendix 2 - Drug-Induced Rhinitis

Cardiovascular

Beta blockers

Alpha blockers

Centrally acting antihypertensives (methyldopa, reserpine)

Angiotensin converting enzyme inhibitors

Niacin

Central Nervous System

Typical/atypical antipsychotics

Chlormethiazole

Citalopram

Gabapentin

Endocrine

Oral contraceptives

Estrogens

Sildenafil

Other

Aspirin/NSAIDs

Mycophenolate mofetil

Penicillamine

Lamivudine
